# Specialised features of melanosomes in health and disease in the retinal pigment epithelium

**DOI:** 10.3389/fcell.2025.1593840

**Published:** 2025-09-03

**Authors:** Dilyana Doncheva, Emily R. Eden, Clare E. Futter

**Affiliations:** UCL Institute of Ophthalmology, University College London, London, United Kingdom

**Keywords:** retinal pigment epithelium, melanosome, albinism, age-related macular degeneration, melanolipofuscin

## Abstract

This mini-review focuses on melanosome biogenesis, positioning and function in the retinal pigment epithelium (RPE) where melanosomes absorb light scatter and protect against the harmful effects of photo-oxidation. RPE melanosomes share a common biogenesis pathway with those of skin melanocytes but are made primarily embryonically and are retained by the RPE throughout life. They do however move from the cell body into the apical processes which, in mammalian RPE, is regulated by a machinery related to that regulating melanosome distribution in skin melanocytes. Melanosomes in the RPE make extensive membrane contacts with the ER and mitochondria although their role in adult RPE remains to be fully established. Albinism is associated with multiple visual defects and reduced or absent pigmentation in melanosomes has implications for long term visual health. Age-related changes in melanosomes have been implicated in retinal degenerative disease, including age-related macular disease (AMD). The lysosomes of the RPE have an unparalleled degradative burden arising from the daily phagocytosis of the distal tips of photoreceptor outer segments, which is part of a daily process of outer segment renewal. A failure to fully process the phagocytosed outer segments leads to a build-up of the toxic ageing pigment, lipofuscin, which accumulates in all ageing RPE. Melanolipofuscin also accumulates in the RPE with age and may result from melanin-mediated degradation of lipofuscin through melanin chemiexcitation. Age-related loss of melanosome-mediated protection could be an important component of age-related visual decline.

## 1 Introduction

**FIGURE 1:**
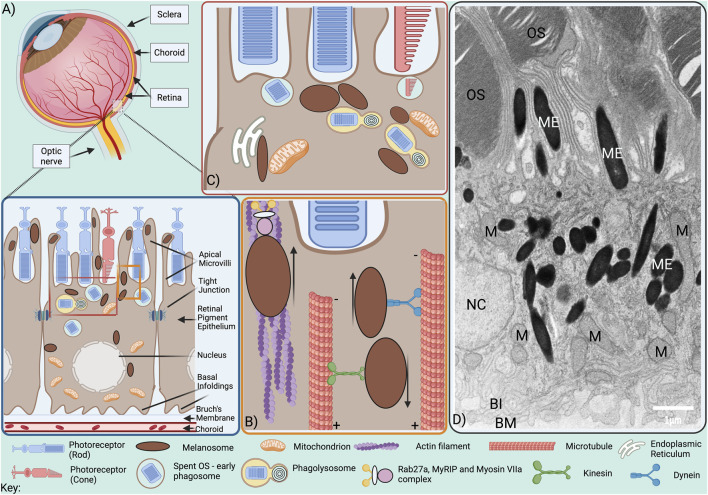
Melanosome positioning within the RPE. **(A)** The RPE is situated at the back of the eye, lying at the retina-choroid interface. At its apical surface, microvilli protrude from RPE cells between rod and cone photoreceptor outer segments (OS), while at the basal surface, the plasma membrane forms infoldings that expand the surface area available for exchange with the highly vascularised choroid, from which it is separated by the supportive matrix of the Bruch’s membrane. Tight junctions are present on RPE lateral borders and while some baso-lateral mitochondria are tethered to the basal infoldings, others are transported to apical RPE following light exposure. Melanosomes are located throughout the cytoplasm and are also transported to apical RPE following light exposure, from where they undergo actin-dependent traffc into the apical processes between OS. **(B)** Minus end-directed dynein-dependent transport along microtubules delivers melanosomes to the actin-rich apical surface from where they are transported into the apical processes by tripartite protein complexes comprised of Rab27a, MyRIP and Myosin VIIa via the actin cytoskeleton. **(C)** Melanosomes interact with mitochondria, phagolysosomes and the ER. **(D)** Electron microscopy image of adult mouse RPE; OS, photoreceptor outer segments; ME, melanosomes; M, mitochondria; NC, nucleus; BI, basal infoldings; BM, Bruch’s membrane. Scale bar, 1 µm.

The retinal pigment epithelium (RPE) forms a monolayer of highly pigmented postmitotic cells at the back of the eye that is central to photoreceptor health, retinal homeostasis and normal visual function. RPE cells are polarised, interfacing apically with the neuroretina and basally with the highly vascularised choroid via a connective tissue layer called Bruch’s membrane (Figure 1A). Thus, the RPE forms part of the blood retinal barrier and regulates transport between the neuroretina and choroidal blood supply. Apically positioned tight junctions help maintain the integrity of the monolayer and enable the RPE to regulate transport between the choroidal blood supply and the neuroretina.

Light-sensing photoreceptors are subject to damage and require continuous renewal of their outer segments (OS) to maintain viability. In humans, OS undergo complete renewal approximately every 11 days, with the damaged OS tips being phagocytosed by the RPE (Lakkaraju et al., 2020; Strauss, 2005). Long RPE apical processes interdigitate the OS, providing mechanical support and assisting with OS phagocytosis. OS regeneration and phagocytosis are precisely coordinated between the RPE and photoreceptors and under circadian control. Phagocytosed OS fuse with endocytic organelles in the RPE for recycling of essential material to the photoreceptor prior to lysosomal delivery for degradation. OS are packed with membranous disks and their daily phagocytosis places a huge metabolic burden on the RPE. With age, autofluorescent granules derived from poorly digested OS become deposited as lipofuscin, accumulation of which is associated with retinal degeneration (Sparrow and Boulton, 2005; Kim et al., 2021).

The RPE also plays an important role in the visual cycle. In response to light, opsin-bound 11-cis-retinal in photoreceptors becomes isomerised to all-trans retinal, activating opsin and triggering phototransduction. However, all-trans-retinol needs to be re-isomerised to 11-cis-retinal in the RPE, to ensure the continuous availability of 11-cis retinal and photoreceptor excitability (Kono et al., 2008; Tsin et al., 2018).

The RPE delivers nutrients and ions from the blood to the neuroretina and electrolytes, water and waste products from the subretinal space to the choroid and regulates the ion composition of the subretinal space to maintain photoreceptor excitability (Strauss, 2005; Lakkaraju et al., 2020). The RPE secretes neuroprotective growth factors like pigment epithelial growth factor (PEDF) (Barnstable and Tombran-Tink, 2004). Basal secretion of low amounts of vascular endothelial growth factor (VEGF) prevents choroidal endothelial cell apoptosis (Strauss, 2005; Apte et al., 2019).

Melanosomes provide photoprotection to both skin and eyes by absorbing light and UV-radiation and scavenging free radicals and reactive oxygen species (Kaufmann and Han, 2024; Seagle et al., 2005b). In the RPE melanosomes also absorb light scatter. Light energy passing through the photoreceptors is captured by apical RPE melanosomes, with melanosome distribution regulated, at least in part, by the light cycle.

A failure of any of the above RPE functions can result in photoreceptor degeneration, retinal dysfunction and concomitant visual loss.

## 2 Melanosomes of the RPE

### 2.1 Melanosome biogenesis

Melanosomes are lysosome-related organelles derived from the endocytic pathway. Studies in skin melanocytes have identified a series of melanosome maturation stages (I–IV) (Seiji et al., 1963; Raposo et al., 2001). Striated amyloid fibril formation is initiated by sorting of the transmembrane premelanosome protein (PMEL) onto intraluminal vesicles of multivesicular endosomes (Stage I) where it is cleaved to form fibrils organized into arrays of parallel sheets, giving melanosomes their ellipsoidal shape (stage II). Delivery of melanin-synthesizing enzymes to the immature melanosome initiates melanin deposition onto the fibrils (Stage III) which are obscured by melanin pigment in mature, Stage IV melanosomes. Although low levels of melanin synthesis may occur in adult RPE (Schraermeyer, 1993), melanosome biogenesis in the RPE is predominantly prenatal, when it proceeds through the same morphologically identifiable stages (I-IV) as in the skin (Lopes et al., 2007b). By day 36 of human embryonic development, the RPE has developed its characteristic pigmentation (Wang et al., 2024) and these melanosomes are retained in the RPE throughout life in contrast to continuous melanosome biogenesis in melanocytes. Melanosome biogenesis is subject to regulation by microphthalmia-associated transcription factor (MITF), a key transcription factor for expression of melanogenic enzymes (Kawakami and Fisher, 2017). Another transcription factor, Myelin regulatory factor (Myrf), has also recently been implicated in regulating melanosome biogenesis, as well as RPE development (Brinkmeier et al., 2025), but to the best of our knowledge, the mechanism for switching off melanosome biogenesis as RPE develop remains elusive. Notably, *in vitro* RPE cell culture models have been published to study melanosome biogenesis, including murine retinal stem cell–derived (Aruta et al., 2011), and human fetal RPE (Boulton, 2014). These, along with disease-specific iPSC-derived RPE (e.g., OCA1A and 2) (George et al., 2022), iron-stimulated ARPE19 cells (Wolkow et al., 2014) and sericin-treated primary human RPE (Eidet et al., 2016) have demonstrated distinct stages of melanosome maturation (I–IV), making them possible systems for studying melanogenesis in the RPE.

### 2.2 Albinism and visual function

Melanin is derived from tyrosinase-mediated oxidation of tyrosine, which, in the presence of cysteine gives rise to pheomelanin (yellow-red) or through tyrosinase-related proteins 1 and 2 to eumelanin (brown-black). Loss of function of melanin synthesizing enzymes results in oculocutaneous albinism (OCA), characterized in the eye by foveal hypoplasia, photophobia, nystagmus, reduction in uncrossed retinal projections and reduced visual acuity (Jeffery, 1998; Oetting and King, 1999) (Table 1). The same characteristics are shared by disorders caused by defective melanosome biogenesis, which are also characterized by functional defects in other lysosome-related organelles, such as platelet dense granules and lytic granules of cytotoxic T lymphocytes (Table 1). Paradoxically, the developmental visual defects result from alterations in neurons that do not produce melanin. A bi-product of melanin synthesis, L-DOPA, is a ligand for GPR143 (OA1) (Lopez et al., 2008), a heterotrimeric G protein-coupled receptor, mutations in which are responsible of ocular albinism type-1, which is characterized at the cellular level by a reduced number of enlarged melanosomes (Incerti et al., 2000). GPR143 signaling within the RPE regulates melanosome motility, interactions with mitochondria, endosome:lysosome fusion, exosome release and secretion of VEGF and PEDGF (Palmisano et al., 2008; Burgoyne et al., 2013; Locke et al., 2014; Lopez et al., 2008). Effects on exosome and cytokine secretion provide a possible explanation of the developmental defects in neurons in ocular albinism.

**TABLE 1:** Examples of genetic pigmentation disorders in the RPE.

Name of disorder	Disorder characteristics	Gene name	Gene function and disease impact on RPE melanosomes	References OMIM
Oculocutaneous Albinism Type 1	Hypopigmentation of the skin, hair and eyes; decreased visual acuity and nystagmus	*TYR*	Tyrosinase, rate-limiting enzyme for melanin synthesis → total loss of RPE melanosomes	Oetting and King (1999) #606933
Usher Syndrome 1B	Progressive retinal degeneration, profound deafness and vestibular defects	*MYO7A*	Myosin7a, actin motor that regulates melanosome motility → lack of melanosomes in apical processes and abnormal phagocytosis	Williams (2008), Weil et al. (1995) #276900
Griscelli Syndrome type 2	Pigmentary dilution of the skin and hair, accumulation of melanosomes in melanocytes and severe haemophagocytic syndrome	*RAB27a*	Rab27a, Rab GTPase that regulates melanosome distribution →lack of melanosomes in apical processes	Van Gele et al. (2009), Futter et al. (2004), Menasche et al. (2000) #607624
Ocular Albinism type 1 (also known as X-linked Ocular Albinism)	Severe developmental ocular defects, including foveal hypoplasia and misrouting of the optic tracts at the chiasm, decreased visual acuity, nystagmus, photophobia and loss of stereoscopic vision	*GPR143*	GPR143 (OA1), G protein coupled receptor that regulates melanosome biogenesis and motility →reduced number of abnormally large melanosomes	Schiaffino (2010), Bassi et al. (1995) #300500
Donnai-Barrow Syndrome	High-grade myopia, large protruding eyes, retinal dystrophy, retinal detachment	*LRP2*	Megalin which regulates the endocytic machinery →abnormally shaped macromelanosomes	Christensen and Birn (2002), Storm et al. (2019) # 222448
Chediak-Higashi Syndrome	Hypopigmentation or oculocutaneous albinism with low vision, nystagmus, and photophobia, and severe immunologic deficiency with neutropenia	*LYST*	LYST that regulates lysosomal trafficking and autophagosome formation →abnormal enlarged lysosomes and melanosomes	Fukai et al. (1996), Weil et al. (1995) #214500
Hermansky-Pudlak Syndrome 7 (HPS-7)	Oculocutaneous albinism, prolonged bleeding (abnormalities in platelet aggregation), pulmonary fibrosis	*DTNBP1*	Dysbindin, component of Biogenesis of Lysosomal Organelle Complex 1 (BLOC-1) →reduced number of irregularly shaped and small RPE melanosomes	Li et al. (2003), Romano et al. (2020) # 614076

### 2.3 Melanosome positioning in the RPE

Melanosome transport to the melanocyte periphery for release from dendrites and subsequent uptake into keratinocytes is key for the photoprotective role of melanin in the skin. Similarly, melanosome transport into apical processes of the RPE may offer photoprotection to the retina. RPE cells, like melanocytes, use motor proteins to transport melanosomes along polar microtubules to the cell periphery, but the microtubular organization in RPE cells differs from that in melanocytes. Whereas the microtubule-organizing centre (MTOC), that anchors microtubule minus-ends, is perinuclear in melanocytes, RPE cells have more apically positioned MTOCs (Jiang et al., 2020). Melanosomes transported along microtubules to the apical surface of mammalian RPE cells are transferred from microtubule motors to the actin motor protein Myosin VIIa (Myosin Va in melanocytes) for tethering to the actin cytoskeleton (Figure 1B) (Gibbs et al., 2004). This process, known as cooperative capture, involves tripartite protein complexes that link Myosin VIIa to Rab27a, a small GTPase on the melanosome surface, via the Rab27a effector MyRIP/Slac2-c (Mlph/Slac2-a in melanocytes) (Lopes et al., 2007a; Futter et al., 2004; Gibbs et al., 2004). In RPE cells from Rab27a-defective ashen and Myosin VIIa-mutant shaker-1 mice, melanosome transport along microtubules was rapid and bidirectional, and melanosomes failed to sequester in the apical region, while disrupting microtubule polymerisation with nocodazole blocked melanosome movement altogether, demonstrating the essential role of microtubule-dependent transport in melanosome positioning (Gibbs et al., 2004; Lopes et al., 2007a).

Bidirectional melanosome transport along microtubules is achieved by recruitment of specific motor proteins, with retrograde minus-end directed transport mediated by dynein motors and anterograde plus-end directed transport towards by kinesin motors (Barral and Seabra, 2004). Due to the difference in polarity, dynein-dependent transport delivers mature melanosomes to the actin-rich apical domain of RPE cells (Figure 1B), but promotes perinuclear positioning in melanocytes (Jiang et al., 2020). The essential role of kinesin-dependent transport to the melanocyte periphery has been challenged, with actin-based melanosome transport through Rab27a interaction with actin nucleator SPIRE1 found to mediate peripheral melanosome positioning in melanocytes (Alzahofi et al., 2020). In contrast, in RPE cells dynein-dependent transport along microtubules is required to deliver melanosomes to the actin-rich apical region (Jiang et al., 2020).

### 2.4 Melanosome positioning defects in disease

Melanosome mislocalisation has been reported in human disease (Table 1). Griscelli syndrome (GS) is a rare autosomal recessive disorder, with the most common form, GS2, caused by RAB27A gene mutations, resulting in impaired melanosome transport to the apical processes of the RPE (Futter et al., 2004). However, in addition to its role in melanosome positioning, Rab27a is important for cytotoxic T-lymphocyte granule release, thus as well as albinism, GS2 patients develop immunodeficiency which can result in severe T-cell and macrophage activation and a much reduced life expectancy (Bizario et al., 2004).

Loss of Rab27a function is also associated with the rare inherited disease choroideraemia. Characterised by progressive vision loss, choroideraemia is caused by defective Rab Escort Protein 1 (REP1) which is involved in Rab GTPase prenylation, a post-translational modification required for Rab protein function (Seabra, 1996). In RPE lacking functional REP1, melanosome transport into the apical processes is impaired, but since degradation of photoreceptor OS is also impaired, the contribution of melanosome mislocalisation to disease pathogenesis and vision loss is not clear (Wavre-Shapton et al., 2013).

Similarly in Usher syndrome 1B, melanosome localisation at the apical region of the RPE is defective due to mutations in the MYO-7A gene, but the significance of melanosome mislocalisation to disease pathology is again unclear since myosin-VIIa also participates in delivery of phagocytosed OS in the RPE for lysosomal degradation (Gibbs et al., 2003). Myosin-VIIa plays additional important roles in the sensory cilia of the inner ear and Usher Syndrome 1B patients are typically profoundly deaf at birth, with progressive retinal degeneration (Ahmed et al., 2013).

### 2.5 Melanosome membrane contact sites (MCS)

Melanosomes are derived from endosomes which, like melanosomes, are transported along microtubules. The loading of microtubule motors onto endosomes is strongly influenced by endosome interaction with the ER at membrane contact sites (MCS), where neighbouring organelles are tethered in close apposition (typically 5–40 nm apart). ER:endosome MCS can both promote kinesin-loading for microtubule plus-end directed endosome movement and prevent dynein interaction with endosomal RILP, an effector protein of endosomal Rab7, thereby reducing minus-end directed movement towards the MTOC (Raiborg et al., 2015). We recently identified extensive ER:melanosome MCS in RPE cells (Burgoyne et al., 2025), but their role in melanosome positioning is yet to be established. Interestingly the ER forms MCS with melanosome of all stages (I-IV) in melanocytes, suggesting additional roles in melanosome maturation (Burgoyne et al., 2025), perhaps orchestrating coordination of maturation with traffic to the periphery for secretion from the dendrites (Wasmeier et al., 2008).

Melanosomes also form MCS with mitochondria, which, in melanocytes, regulate melanosome biogenesis, likely through ATP provision (Daniele et al., 2014). Melanosome biogenesis occurs in the perinuclear region of melanocytes, where mitochondrial MCS were found to be most abundant and is stimulated by GPR143, which also promotes melanosome:mitochondria MCS. The mitochondrial protein mitofusin-2 is involved in tethering to melanosomes and required for the stimulation of melanosome biogenesis by GPR143. Melanosomes also form MCS with mitochondria in mature RPE (Figures 1C,D) (Burgoyne et al., 2025; Neto et al., 2024), suggesting additional functions, possibly in scavenging mitochondrial ROS (Seagle et al., 2005a), or in Ca^2+^ flux between the two organelles as has been shown for mitochondria:lysosome MCS (Peng et al., 2020) and implicated in melanosome acidification (Ambrosio et al., 2016). Tripartite ER:melanosome:mitochondria MCS found in the RPE may further facilitate buffering of cytosolic Ca^2+^ and quenching of ROS (Burgoyne et al., 2025).

## 3 RPE melanosomes in ageing and retinal degeneration

### 3.1 Age-related changes in RPE melanosomes

Unlike melanosomes of the skin, RPE melanosomes are long-lived. Ageing is, however, accompanied by reduced melanosome numbers and total melanin within the RPE (Feeney-Burns et al., 1984; Sarna et al., 2003). The melanin becomes more irregularly shaped and less compact and in individuals over 90 years of age virtually all melanosomes are surrounded by other material, including lipofuscin (Feeney-Burns et al., 1990). Formation of melanolipofuscin, an ill-defined complex, could contribute to the reduced melanosome numbers in the RPE, but does not explain the reduction in total melanin. Melanin polymer is resistant to enzymatic degradation but exposure to intense light, ultraviolet light or oxidising agents can induce RPE melanin degradation (Zadlo et al., 2007; Dontsov et al., 2017). The high levels of light or UV light required to degrade melanin would be unlikely to be encountered by the RPE *in vivo* but large amounts of superoxides and other reactive oxygen species (ROS) can be produced by lipofuscin-containing granules on light exposure. Direct contact between melanin and lipofuscin within melanolipofuscin granules thus creates conditions favourable for melanin degradation. This leads to a reduction in light absorbance, and anti-oxidant and anti-radical functions, and an increase in melanin’s photochemical reactivity (Olchawa et al., 2021; Zadlo et al., 2007). Thus, melanin degradation can transform melanin-containing granules from “protective” to “toxic”. The association between melanin and lipofuscin in melanolipofuscin has the potential not only to promote melanin degradation, but also to promote lipofuscin degradation by melanin chemiexcitation (see below).

### 3.2 Role of melanosomes in limiting lipofuscin accumulation

#### 3.2.1 Lipofuscin accumulation

The RPE has a huge degradative burden over a typical lifespan and lipofuscin accumulation begins at an early age (Feeney-Burns et al., 1984). Lipofuscin accumulates in lysosomes and, indeed, lysosomal delivery of OS was shown to promote the development of autofluorescent lipofuscin-like granules in cultured RPE (Escrevente et al., 2021). Lipofuscin accumulation can elevate lysosomal pH, inhibiting proteases and lipases, and inhibit cholesterol efflux (Lakkaraju et al., 2007; Finnemann et al., 2002; Shamsi and Boulton, 2001). In the presence of light and oxygen, bisretinoids within lipofuscin can be oxidised forming toxic products that remain in the lipofuscin granule or leak into the cytoplasm causing photo-oxidative stress that can also lead to inflammation and, ultimately, cell death (Dontsov and Ostrovsky, 2024). Stargardt’s disease caused by loss of function of the lipid flippase, ABCA4 leads to premature and exaggerated accumulation of lipofuscin in the RPE accompanied by degeneration of the neural retina and visual decline (Molday, 2007). Lipofuscin accumulation in Stargardt’s disease mouse models is increased on an albino background supporting a role for melanin in limiting lipofuscin accumulation (Taubitz et al., 2018).

#### 3.2.2 Melanolipofuscin accumulation

The name “Melanolipofuscin” implies that it arises from lipofuscin-containing granules associating/fusing with melanosomes. The presence of autophagy markers and absence of photoreceptor proteins in melanolipofuscin led one study to conclude that melanolipofuscin is formed by melanosome autophagy (Warburton et al., 2007). However, intact melanosomes frequently associate with phagosomes (Neto et al., 2024; Wavre-Shapton et al., 2014) and gold particles labelling OS injected into the subretinal space of rats were subsequently recovered in RPE melanosomes, demonstrating a connection between the phagocytic pathway and melanosomes (Schraermeyer et al., 1999). Furthermore, in pigmented mouse models of Stargardt’s disease and Choroideremia, lipofuscin accumulation/defects in phagocytosed OS processing are accompanied by melanolipofuscin accumulation (Wavre-Shapton et al., 2013; Charbel Issa et al., 2013).

#### 3.2.3 Melanin chemiexcitation

A direct role for melanin in limiting lipofuscin production has been indicated by the reversal of excess lipofuscin accumulation in albino Stargardt’s disease mice by virally-induced tyrosinase expression (Lyu et al., 2023). Tyrosinase expression, whilst sufficient to induce melanin synthesis, does not induce melanosome formation so the melanin produced likely accumulates in lysosomes with ready access to lipofuscin accumulated within the same organelle. Superoxide generators accelerated lipofuscin degradation but only in the presence of melanin through generation of excited electron states on melanin (melanin chemiexcitation) (Lyu et al., 2023). This led to the proposal that OS are partially processed in lysosomes before delivery of lipofuscin to melanosomes for further processing. The trafficking steps and molecular mechanisms required for the meeting of melanin and lipofuscin remain to be elucidated. In ageing RPE irregular shaped melanin is found frequently surrounded by lipofuscin, suggesting fusion between melanosomes and lipofuscin -containing lysosomes. Whether the tethering and SNARE complexes responsible for fusion of lysosomes with other organelles operate in lysosomal fusion with melanosomes remains to be determined, but it is possible that melanosome Ca^2+^ stores (Salceda and Sanchez-Chavez, 2000) could contribute to SNARE-dependent fusion events (Di Giovanni et al., 2010). The role of autophagy in generation of melanolipofuscin also requires further investigation. Importantly chemi-excitation of melanin results in its destruction, contributing to the age-related loss of ‘functional’ melanin (Lyu et al., 2023).

### 3.3 The relationship between pigmentation and AMD

AMD is the greatest cause of registered blindness in the developed world and is a multifactorial disease with genetic and environmental risk factors, but the greatest risk is age. AMD is characterised by accumulation of lipid-rich deposits basal to the RPE (Drusen) and apical to the RPE (pseudoDrusen). Dry AMD can progress to loss of photoreceptors and RPE cells, known as geographic atrophy, and wet AMD can progress to neovascularisation that compromises Bruchs membrane and the RPE monolayer.

#### 3.3.1 A protective role for RPE melanin in AMD?

As described above, melanin can limit lipofuscin accumulation in the RPE. Interestingly, the accumulation of melanolipofuscin more closely reflects the age of onset of AMD than lipofuscin alone (Feeney-Burns, 1980). Caucasians are significantly more susceptible to AMD than those of African descent (Klein et al., 2006; Klein et al., 2013; Wong et al., 2014). A protective role for melanin in AMD would predict that albinism would be linked with a high AMD risk. This has not to our knowledge been reported but could be because albinism is relatively rare and sufferers have a low visual acuity from an early age potentially hindering recognition of AMD. Genome-wide association studies have identified gene variants associated with AMD risk, including complement pathway, extracellular matrix and lipid metabolism genes, the latter including apolipoprotein E (APO-E) (Fritsche et al., 2016; Holliday et al., 2013). APO-E is required for sorting and processing of PMEL within immature melanosomes and the resulting generation of striations on which melanin is deposited (van Niel et al., 2015) but whether APO-E gene variants carrying AMD risk are compromised in this function is unclear. Human patients and animal models with lysosomal storage diseases caused by loss of function of specific lysosomal components frequently exhibit AMD-like features. For example, mutation of the lysosomal protease, cathepsin D, causes an aggressive form of the neurodegenerative Batten’s disease and Cathepsin D mutant mice accumulate autofluorescent material in the RPE as well as extracellular basal deposits (Rakoczy et al., 2002). In Danon disease, caused by loss of function of the lysosomal membrane protein LAMP2, autofluorescent material accumulates in the RPE, accompanied by basal deposits and eventual photoreceptor and RPE loss (Notomi et al., 2019). A systematic investigation of the effects of defects in melanosome biogenesis or movement on the phenotype of lysosomal storage disease models would help to elucidate the role of melanosomes in the development of AMD features.

#### 3.3.2 GPR143 and AMD

The GPR143 ligand, L-DOPA, is produced during melanin synthesis and Parkinson’s disease patients taking L-DOPA showed reduced/delayed onset of AMD (Brilliant et al., 2016), suggesting that signalling from GPR143 might protect from AMD. Elevated GPR143 signalling enhances PEGF secretion and suppresses VEGF secretion which could contribute to the protective effect of L-DOPA on risk of geographic atrophy and neovascularisation (Tung and McKay, 2023). It has also been proposed that signalling from GPR143 could be key to the potential protective effect of melanin on AMD (Tung and McKay, 2023). Given that most melanin synthesis is prenatal in the RPE, the source of L-DOPA in ageing RPE is not clear and may not be related to melanosomes *per se*.

#### 3.3.3 Melanin transfer to mononuclear phagocytes in AMD

The appearance of hyperreflective foci in spectral domain optical coherence tomography (SD-OCT) images can predict progression to late stage AMD (Leuschen et al., 2013; Christenbury et al., 2013). Although originally thought to be floating RPE cells they were recently shown to be mononuclear phagophores containing melanin (Augustin et al., 2023), raising the question of how they acquired melanin when RPE cells do not normally secrete it. One possibility could be phagocytosis of dying RPE cells. Interestingly CD147 knockout mice have more hyperreflective foci, and reduced RPE pigmentation without RPE cell loss. The authors propose that CD147 acts as a “do not eat me” signal on the RPE that when lost results in phagocytosis of melanosome-containing RPE apical processes that contain melanosomes (Augustin et al., 2023). The trafficking events leading to melanin transfer from RPE cells to phagophore remain to be elucidated and, as with the transfer of melanin from skin melanocytes to keratinocytes, more than one mechanism is possible. In skin the predominant mechanism is melanosome fusion with the plasma membrane and melanin secretion (Benito-Martinez et al., 2021; Bento-Lopes et al., 2023). CD147 levels on the RPE decline with age and AMD, and so loss of melanin to MPs could contribute to the age-related decline in RPE pigmentation and increased risk of AMD progression.

## 4 Perspective

Their long-lived nature and lifelong exposure to photo-oxidative stress distinguishes melanosomes of the RPE from those of skin melanocytes and raises questions about their roles in RPE-specific functions and retinal degenerative disease. What regulates melanosome:phagosome interactions and what is their role in processing phagocytosed OS and generating melanolipofuscin? What is the role of age-related changes in melanin in retinal degenerative diseases like AMD? What is the function of the numerous membrane contacts between melanosomes and other organelles in the RPE and how are they regulated? Increased understanding of melanosome function may pave the way for novel melanosome-targeted therapeutic approaches to the many diseases associated with melanosome misregulation.

## References

[B1] Ahmed Z. M. Frolenkov G. I. Riazuddin S. (2013). Usher proteins in inner ear structure and function. Physiol. Genomics 45, 987–989. 10.1152/physiolgenomics.00135.2013 24022220 PMC3841788

[B2] Alzahofi N. Welz T. Robinson C. L. Page E. L. Briggs D. A. Stainthorp A. K. (2020). Rab27a co-ordinates actin-dependent transport by controlling organelle-associated motors and track assembly proteins. Nat. Commun. 11, 3495. 10.1038/s41467-020-17212-6 32661310 PMC7359353

[B3] Ambrosio A. L. Boyle J. A. Aradi A. E. Christian K. A. DI Pietro S. M. (2016). TPC2 controls pigmentation by regulating melanosome pH and size. Proc. Natl. Acad. Sci. U. S. A. 113, 5622–5627. 10.1073/pnas.1600108113 27140606 PMC4878521

[B4] Apte R. S. Chen D. S. Ferrara N. (2019). VEGF in signaling and disease: beyond discovery and development. Cell 176, 1248–1264. 10.1016/j.cell.2019.01.021 30849371 PMC6410740

[B5] Aruta C. Giordano F. De Marzo A. Comitato A. Raposo G. Nandrot E. F. (2011). *In* vitro differentiation of retinal pigment epithelium from adult retinal stem cells. Pigment. Cell Melanoma Res. 24, 233–240. 10.1111/j.1755-148X.2010.00793.x 21232026

[B6] Augustin S. Lam M. Lavalette S. Verschueren A. Blond F. Forster V. (2023). Melanophages give rise to hyperreflective foci in AMD, a disease-progression marker. J. Neuroinflammation 20, 28. 10.1186/s12974-023-02699-9 36755326 PMC9906876

[B7] Barnstable C. J. Tombran-Tink J. (2004). Neuroprotective and antiangiogenic actions of PEDF in the eye: molecular targets and therapeutic potential. Prog. Retin Eye Res. 23, 561–577. 10.1016/j.preteyeres.2004.05.002 15302351

[B8] Barral D. C. Seabra M. C. (2004). The melanosome as a model to study organelle motility in mammals. Pigment. Cell Res. 17, 111–118. 10.1111/j.1600-0749.2004.00138.x 15016299

[B9] Bassi M. T. Schiaffino M. V. Renieri A. De Nigris F. Galli L. Bruttini M. (1995). Cloning of the gene for ocular albinism type 1 from the distal short arm of the X chromosome. Nat. Genet. 10, 13–19. 10.1038/ng0595-13 7647783

[B10] Benito-Martinez S. Salavessa L. Raposo G. Marks M. S. Delevoye C. (2021). Melanin transfer and fate within keratinocytes in human skin pigmentation. Integr. Comp. Biol. 61, 1546–1555. 10.1093/icb/icab094 34021340 PMC8516110

[B11] Bento-Lopes L. Cabaco L. C. Charneca J. Neto M. V. Seabra M. C. Barral D. C. (2023). Melanin’s journey from melanocytes to keratinocytes: uncovering the molecular mechanisms of melanin transfer and processing. Int. J. Mol. Sci. 24, 11289. 10.3390/ijms241411289 37511054 PMC10379423

[B12] Bizario J. C. Feldmann J. Castro F. A. Menasche G. Jacob C. M. Cristofani L. (2004). Griscelli syndrome: characterization of a new mutation and rescue of T-cytotoxic activity by retroviral transfer of RAB27A gene. J. Clin. Immunol. 24, 397–410. 10.1023/B:JOCI.0000029119.83799.cb 15163896

[B13] Boulton M. E. (2014). Studying melanin and lipofuscin in RPE cell culture models. Exp. Eye Res. 126, 61–67. 10.1016/j.exer.2014.01.016 25152361 PMC4143628

[B14] Brilliant M. H. Vaziri K. Connor T. B. Jr. Schwartz S. G. Carroll J. J. Mccarty C. A. (2016). Mining retrospective data for virtual prospective drug repurposing: L-DOPA and age-related macular degeneration. Am. J. Med. 129, 292–298. 10.1016/j.amjmed.2015.10.015 26524704 PMC4841631

[B15] Brinkmeier M. L. Wang S. Q. Pittman H. A. Cheung L. Y. Prasov L. (2025). Myelin regulatory factor (MYRF) is a critical early regulator of retinal pigment epithelial development. PLoS Genet. 21, e1011670. 10.1371/journal.pgen.1011670 40233131 PMC12052213

[B16] Burgoyne T. Jolly R. Martin-Martin B. Seabra M. C. Piccirillo R. Schiaffino M. V. (2013). Expression of OA1 limits the fusion of a subset of MVBs with lysosomes - a mechanism potentially involved in the initial biogenesis of melanosomes. J. Cell Sci. 126, 5143–5152. 10.1242/jcs.128561 24006264 PMC3828590

[B17] Burgoyne T. Doncheva D. Eden E. R. (2025). Identification of ER:melanosome membrane contact sites in the retinal pigment epithelium. bioRxiv 8. 2025.01.07.631750. 10.1177/25152564251340949 40463419 PMC12130655

[B18] Charbel Issa P. Barnard A. R. Singh M. S. Carter E. Jiang Z. Radu R. A. (2013). Fundus autofluorescence in the Abca4(-/-) mouse model of Stargardt disease--correlation with accumulation of A2E, retinal function, and histology. Invest Ophthalmol. Vis. Sci. 54, 5602–5612. 10.1167/iovs.13-11688 23761084 PMC3747716

[B19] Christenbury J. G. Folgar F. A. O’Connell R. V. Chiu S. J. Farsiu S. Toth C. A. (2013). Progression of intermediate age-related macular degeneration with proliferation and inner retinal migration of hyperreflective foci. Ophthalmology 120, 1038–1045. 10.1016/j.ophtha.2012.10.018 23352193 PMC3640702

[B20] Christensen E. I. Birn H. (2002). Megalin and cubilin: multifunctional endocytic receptors. Nat. Rev. Mol. Cell Biol. 3, 256–266. 10.1038/nrm778 11994745

[B21] Daniele T. Hurbain I. Vago R. Casari G. Raposo G. Tacchetti C. (2014). Mitochondria and melanosomes establish physical contacts modulated by Mfn2 and involved in organelle biogenesis. Curr. Biol. 24, 393–403. 10.1016/j.cub.2014.01.007 24485836

[B22] Di Giovanni J. Iborra C. Maulet Y. Leveque C. El Far O. Seagar M. (2010). Calcium-dependent regulation of SNARE-mediated membrane fusion by calmodulin. J. Biol. Chem. 285, 23665–23675. 10.1074/jbc.M109.096073 20519509 PMC2911300

[B23] Dontsov A. Ostrovsky M. (2024). Retinal pigment epithelium pigment granules: norms, age relations and pathology. Int. J. Mol. Sci. 25, 3609. 10.3390/ijms25073609 38612421 PMC11011557

[B24] Dontsov A. E. Sakina N. L. Ostrovsky M. A. (2017). Loss of melanin by eye retinal pigment epithelium cells is associated with its oxidative destruction in melanolipofuscin granules. Biochem. (Mosc) 82, 916–924. 10.1134/S0006297917080065 28941459

[B25] Eidet J. R. Reppe S. Pasovic L. Olstad O. K. Lyberg T. Khan A. Z. (2016). The silk-protein sericin induces rapid melanization of cultured primary human retinal pigment epithelial cells by activating the NF-κB pathway. Sci. Rep. 6, 22671. 10.1038/srep22671 26940175 PMC4778122

[B26] Escrevente C. Falcao A. S. Hall M. J. Lopes-Da-Silva M. Antas P. Mesquita M. M. (2021). Formation of lipofuscin-like autofluorescent granules in the retinal pigment epithelium requires lysosome dysfunction. Invest Ophthalmol. Vis. Sci. 62, 39. 10.1167/iovs.62.9.39 34313720 PMC8322709

[B27] Feeney-Burns L. (1980). The pigments of the retinal pigment epithelium. Curr. Top. Eye Res. 2, 119–178. 6807609

[B28] Feeney-Burns L. Hilderbrand E. S. Eldridge S. (1984). Aging human RPE: morphometric analysis of macular, equatorial, and peripheral cells. Invest Ophthalmol. Vis. Sci. 25, 195–200. 6698741

[B29] Feeney-Burns L. Burns R. P. Gao C. L. (1990). Age-related macular changes in humans over 90 years old. Am. J. Ophthalmol. 109, 265–278. 10.1016/s0002-9394(14)74549-0 2309857

[B30] Finnemann S. C. Leung L. W. Rodriguez-Boulan E. (2002). The lipofuscin component A2E selectively inhibits phagolysosomal degradation of photoreceptor phospholipid by the retinal pigment epithelium. Proc. Natl. Acad. Sci. U. S. A. 99, 3842–3847. 10.1073/pnas.052025899 11904436 PMC122611

[B31] Fritsche L. G. Igl W. Bailey J. N. Grassmann F. Sengupta S. Bragg-Gresham J. L. (2016). A large genome-wide association study of age-related macular degeneration highlights contributions of rare and common variants. Nat. Genet. 48, 134–143. 10.1038/ng.3448 26691988 PMC4745342

[B32] Fukai K. Oh J. Karim M. A. Moore K. J. Kandil H. H. Ito H. (1996). Homozygosity mapping of the gene for Chediak-Higashi syndrome to chromosome 1q42-q44 in a segment of conserved synteny that includes the mouse beige locus (bg). Am. J. Hum. Genet. 59, 620–624. 8751863 PMC1914913

[B33] Futter C. E. Ramalho J. S. Jaissle G. B. Seeliger M. W. Seabra M. C. (2004). The role of Rab27a in the regulation of melanosome distribution within retinal pigment epithelial cells. Mol. Biol. Cell 15, 2264–2275. 10.1091/mbc.e03-10-0772 14978221 PMC404021

[B34] George A. Sharma R. Pfister T. Abu-Asab M. Hotaling N. Bose D. (2022). *In* vitro disease modeling of oculocutaneous albinism type 1 and 2 using human induced pluripotent stem cell-derived retinal pigment epithelium. Stem Cell Rep. 17, 173–186. 10.1016/j.stemcr.2021.11.016 35021041 PMC8758966

[B35] Gibbs D. Kitamoto J. Williams D. S. (2003). Abnormal phagocytosis by retinal pigmented epithelium that lacks myosin VIIa, the Usher syndrome 1B protein. Proc. Natl. Acad. Sci. U. S. A. 100, 6481–6486. 10.1073/pnas.1130432100 12743369 PMC164472

[B36] Gibbs D. Azarian S. M. Lillo C. Kitamoto J. Klomp A. E. Steel K. P. (2004). Role of myosin VIIa and Rab27a in the motility and localization of RPE melanosomes. J. Cell Sci. 117, 6473–6483. 10.1242/jcs.01580 15572405 PMC2942070

[B37] Holliday E. G. Smith A. V. Cornes B. K. Buitendijk G. H. Jensen R. A. Sim X. (2013). Insights into the genetic architecture of early stage age-related macular degeneration: a genome-wide association study meta-analysis. PLoS One 8, e53830. 10.1371/journal.pone.0053830 23326517 PMC3543264

[B38] Incerti B. Cortese K. Pizzigoni A. Surace E. M. Varani S. Coppola M. (2000). Oa1 knock-out: new insights on the pathogenesis of ocular albinism type 1. Hum. Mol. Genet. 9, 2781–2788. 10.1093/hmg/9.19.2781 11092754

[B39] Jeffery G. (1998). The retinal pigment epithelium as a developmental regulator of the neural retina. Eye (Lond) 12 (Pt 3b), 499–503. 10.1038/eye.1998.137 9775209

[B40] Jiang M. Paniagua A. E. Volland S. Wang H. Balaji A. Li D. G. (2020). Microtubule motor transport in the delivery of melanosomes to the actin-rich apical domain of the retinal pigment epithelium. J. Cell Sci. 133, jcs242214. 10.1242/jcs.242214 32661088 PMC7420818

[B41] Kaufmann M. Han Z. (2024). RPE melanin and its influence on the progression of AMD. Ageing Res. Rev. 99, 102358. 10.1016/j.arr.2024.102358 38830546 PMC11260545

[B42] Kawakami A. Fisher D. E. (2017). The master role of microphthalmia-associated transcription factor in melanocyte and melanoma biology. Lab. Invest 97, 649–656. 10.1038/labinvest.2017.9 28263292

[B43] Kim H. J. Montenegro D. Zhao J. Sparrow J. R. (2021). Bisretinoids of the retina: photo-oxidation, iron-catalyzed oxidation, and disease consequences. Antioxidants (Basel) 10, 1382. 10.3390/antiox10091382 34573014 PMC8467448

[B44] Klein R. Klein B. E. Knudtson M. D. Wong T. Y. Cotch M. F. Liu K. (2006). Prevalence of age-related macular degeneration in 4 racial/ethnic groups in the multi-ethnic study of atherosclerosis. Ophthalmology 113, 373–380. 10.1016/j.ophtha.2005.12.013 16513455

[B45] Klein R. Li X. Kuo J. Z. Klein B. E. Cotch M. F. Wong T. Y. (2013). Associations of candidate genes to age-related macular degeneration among racial/ethnic groups in the multi-ethnic study of atherosclerosis. Am. J. Ophthalmol. 156, 1010–1020 e1. 10.1016/j.ajo.2013.06.004 23938121 PMC3812928

[B46] Kono M. Goletz P. W. Crouch R. K. (2008). 11-cis- and all-trans-retinols can activate rod opsin: rational design of the visual cycle. Biochemistry 47, 7567–7571. 10.1021/bi800357b 18563917 PMC2561911

[B47] Lakkaraju A. Finnemann S. C. Rodriguez-Boulan E. (2007). The lipofuscin fluorophore A2E perturbs cholesterol metabolism in retinal pigment epithelial cells. Proc. Natl. Acad. Sci. U. S. A. 104, 11026–11031. 10.1073/pnas.0702504104 17578916 PMC1904145

[B48] Lakkaraju A. Umapathy A. Tan L. X. Daniele L. Philp N. J. Boesze-Battaglia K. (2020). The cell biology of the retinal pigment epithelium. Prog. Retin Eye Res. 78, 100846. 10.1016/j.preteyeres.2020.100846 32105772 PMC8941496

[B49] Leuschen J. N. Schuman S. G. Winter K. P. Mccall M. N. Wong W. T. Chew E. Y. (2013). Spectral-domain optical coherence tomography characteristics of intermediate age-related macular degeneration. Ophthalmology 120, 140–150. 10.1016/j.ophtha.2012.07.004 22968145 PMC3536919

[B50] Li W. Zhang Q. Oiso N. Novak E. K. Gautam R. O'Brien E. P. (2003). Hermansky-Pudlak syndrome type 7 (HPS-7) results from mutant dysbindin, a member of the biogenesis of lysosome-related organelles complex 1 (BLOC-1). Nat. Genet. 35, 84–89. 10.1038/ng1229 12923531 PMC2860733

[B51] Locke C. J. Congrove N. R. Dismuke W. M. Bowen T. J. Stamer W. D. Mckay B. S. (2014). Controlled exosome release from the retinal pigment epithelium in situ. Exp. Eye Res. 129, 1–4. 10.1016/j.exer.2014.10.010 25311167

[B52] Lopes V. S. Ramalho J. S. Owen D. M. Karl M. O. Strauss O. Futter C. E. (2007a). The ternary Rab27a-Myrip-Myosin VIIa complex regulates melanosome motility in the retinal pigment epithelium. Traffic 8, 486–499. 10.1111/j.1600-0854.2007.00548.x 17451552 PMC1920545

[B53] Lopes V. S. Wasmeier C. Seabra M. C. Futter C. E. (2007b). Melanosome maturation defect in Rab38-deficient retinal pigment epithelium results in instability of immature melanosomes during transient melanogenesis. Mol. Biol. Cell 18, 3914–3927. 10.1091/mbc.e07-03-0268 17671165 PMC1995718

[B54] Lopez V. M. Decatur C. L. Stamer W. D. Lynch R. M. Mckay B. S. (2008). L-DOPA is an endogenous ligand for OA1. PLoS Biol. 6, e236. 10.1371/journal.pbio.0060236 18828673 PMC2553842

[B55] Lyu Y. Tschulakow A. V. Wang K. Brash D. E. Schraermeyer U. (2023). Chemiexcitation and melanin in photoreceptor disc turnover and prevention of macular degeneration. Proc. Natl. Acad. Sci. U. S. A. 120, e2216935120. 10.1073/pnas.2216935120 37155898 PMC10194005

[B56] Menasche G. Pastural E. Feldmann J. Certain S. Ersoy F. Dupuis S. (2000). Mutations in RAB27A cause Griscelli syndrome associated with haemophagocytic syndrome. Nat. Genet. 25, 173–176. 10.1038/76024 10835631

[B57] Molday R. S. (2007). ATP-binding cassette transporter ABCA4: molecular properties and role in vision and macular degeneration. J. Bioenerg. Biomembr. 39, 507–517. 10.1007/s10863-007-9118-6 17994272

[B58] Neto M. V. De Rossi G. Berkowitz B. A. Seabra M. C. Luthert P. J. Futter C. E. (2024). Daily light onset and plasma membrane tethers regulate mitochondria redistribution within the retinal pigment epithelium. Cells 13, 1100. 10.3390/cells13131100 38994953 PMC11240580

[B59] Notomi S. Ishihara K. Efstathiou N. E. Lee J. J. Hisatomi T. Tachibana T. (2019). Genetic LAMP2 deficiency accelerates the age-associated formation of basal laminar deposits in the retina. Proc. Natl. Acad. Sci. U. S. A. 116, 23724–23734. 10.1073/pnas.1906643116 31699817 PMC6876195

[B60] Oetting W. S. King R. A. (1999). Molecular basis of albinism: mutations and polymorphisms of pigmentation genes associated with albinism. Hum. Mutat. 13, 99–115. 10.1002/(SICI)1098-1004(1999)13:2<99::AID-HUMU2>3.0.CO;2-C 10094567

[B61] Olchawa M. M. Szewczyk G. M. Zadlo A. C. Krzysztynska-Kuleta O. I. Sarna T. J. (2021). The effect of aging and antioxidants on photoreactivity and phototoxicity of human melanosomes: an in vitro study. Pigment. Cell Melanoma Res. 34, 670–682. 10.1111/pcmr.12914 32702137

[B62] Palmisano I. Bagnato P. Palmigiano A. Innamorati G. Rotondo G. Altimare D. (2008). The ocular albinism type 1 protein, an intracellular G protein-coupled receptor, regulates melanosome transport in pigment cells. Hum. Mol. Genet. 17, 3487–3501. 10.1093/hmg/ddn241 18697795 PMC2572695

[B63] Peng W. Wong Y. C. Krainc D. (2020). Mitochondria-lysosome contacts regulate mitochondrial Ca(2+) dynamics via lysosomal TRPML1. Proc. Natl. Acad. Sci. U. S. A. 117, 19266–19275. 10.1073/pnas.2003236117 32703809 PMC7430993

[B64] Raiborg C. Wenzel E. M. Stenmark H. (2015). ER-endosome contact sites: molecular compositions and functions. EMBO J. 34, 1848–1858. 10.15252/embj.201591481 26041457 PMC4547891

[B65] Rakoczy P. E. Zhang D. Robertson T. Barnett N. L. Papadimitriou J. Constable I. J. (2002). Progressive age-related changes similar to age-related macular degeneration in a transgenic mouse model. Am. J. Pathol. 161, 1515–1524. 10.1016/S0002-9440(10)64427-6 12368224 PMC1867306

[B66] Raposo G. Tenza D. Murphy D. M. Berson J. F. Marks M. S. (2001). Distinct protein sorting and localization to premelanosomes, melanosomes, and lysosomes in pigmented melanocytic cells. J. Cell Biol. 152, 809–824. 10.1083/jcb.152.4.809 11266471 PMC2195785

[B67] Romano G. L. Platania C. B. M. Leggio G. M. Torrisi S. A. Giunta S. Salomone S. (2020). Retinal biomarkers and pharmacological targets for Hermansky-Pudlak syndrome 7. Sci. Rep. 10, 3972. 10.1038/s41598-020-60931-5 32132582 PMC7055265

[B68] Salceda R. Sanchez-Chavez G. (2000). Calcium uptake, release and ryanodine binding in melanosomes from retinal pigment epithelium. Cell Calcium 27, 223–229. 10.1054/ceca.2000.0111 10858668

[B69] Sarna T. Burke J. M. Korytowski W. Rozanowska M. Skumatz C. M. Zareba A. (2003). Loss of melanin from human RPE with aging: possible role of melanin photooxidation. Exp. Eye Res. 76, 89–98. 10.1016/s0014-4835(02)00247-6 12589778

[B70] Schiaffino M. V. (2010). Signaling pathways in melanosome biogenesis and pathology. Int. J. Biochem. Cell Biol. 42, 1094–1104. 10.1016/j.biocel.2010.03.023 20381640 PMC2885761

[B71] Schraermeyer U. (1993). Does melanin turnover occur in the eyes of adult vertebrates? Pigment. Cell Res. 6, 193–204. 10.1111/j.1600-0749.1993.tb00602.x 8248016

[B72] Schraermeyer U. Peters S. Thumann G. Kociok N. Heimann K. (1999). Melanin granules of retinal pigment epithelium are connected with the lysosomal degradation pathway. Exp. Eye Res. 68, 237–245. 10.1006/exer.1998.0596 10068489

[B73] Seabra M. C. (1996). New insights into the pathogenesis of choroideremia: a tale of two REPs. Ophthalmic Genet. 17, 43–46. 10.3109/13816819609057869 8832719

[B74] Seagle B. L. Rezai K. A. Gasyna E. M. Kobori Y. Rezaei K. A. Norris J. R. Jr (2005a). Time-resolved detection of melanin free radicals quenching reactive oxygen species. J. Am. Chem. Soc. 127, 11220–11221. 10.1021/ja052773z 16089432

[B75] Seagle B. L. Rezai K. A. Kobori Y. Gasyna E. M. Rezaei K. A. Norris J. R. JR. (2005b). Melanin photoprotection in the human retinal pigment epithelium and its correlation with light-induced cell apoptosis. Proc. Natl. Acad. Sci. U. S. A. 102, 8978–8983. 10.1073/pnas.0501971102 15951427 PMC1157035

[B76] Seiji M. Fitzpatrick T. B. Simpson R. T. Birbeck M. S. (1963). Chemical composition and terminology of specialized organelles (melanosomes and melanin granules) in mammalian melanocytes. Nature 197, 1082–1084. 10.1038/1971082a0 13992623

[B77] Shamsi F. A. Boulton M. (2001). Inhibition of RPE lysosomal and antioxidant activity by the age pigment lipofuscin. Invest Ophthalmol. Vis. Sci. 42, 3041–3046. 11687553

[B78] Sparrow J. R. Boulton M. (2005). RPE lipofuscin and its role in retinal pathobiology. Exp. Eye Res. 80, 595–606. 10.1016/j.exer.2005.01.007 15862166

[B79] Storm T. Burgoyne T. Dunaief J. L. Christensen E. I. Futter C. Nielsen R. (2019). Selective ablation of Megalin in the retinal pigment epithelium results in Megaophthalmos, macromelanosome formation and severe retina degeneration. Invest Ophthalmol. Vis. Sci. 60, 322–330. 10.1167/iovs.18-25667 30665232 PMC6343679

[B80] Strauss O. (2005). The retinal pigment epithelium in visual function. Physiol. Rev. 85, 845–881. 10.1152/physrev.00021.2004 15987797

[B81] Taubitz T. Tschulakow A. V. Tikhonovich M. Illing B. Fang Y. Biesemeier A. (2018). Ultrastructural alterations in the retinal pigment epithelium and photoreceptors of a Stargardt patient and three Stargardt mouse models: indication for the central role of RPE melanin in oxidative stress. PeerJ 6, e5215. 10.7717/peerj.5215 30038866 PMC6054867

[B82] Tsin A. Betts-Obregon B. Grigsby J. (2018). Visual cycle proteins: structure, function, and roles in human retinal disease. J. Biol. Chem. 293, 13016–13021. 10.1074/jbc.AW118.003228 30002120 PMC6109927

[B83] Tung D. Mckay B. S. (2023). Decoding race and age-related macular degeneration: GPR 143 activity is the key. Adv. Exp. Med. Biol. 1415, 43–47. 10.1007/978-3-031-27681-1_7 37440012

[B84] Van Gele M. Dynoodt P. Lambert J. (2009). Griscelli syndrome: a model system to study vesicular trafficking. Pigment. Cell Melanoma Res. 22, 268–282. 10.1111/j.1755-148X.2009.00558.x 19243575

[B85] Van Niel G. Bergam P. DI Cicco A. Hurbain I. Lo Cicero A. Dingli F. (2015). Apolipoprotein E regulates amyloid formation within endosomes of pigment cells. Cell Rep. 13, 43–51. 10.1016/j.celrep.2015.08.057 26387950

[B86] Wang S. Li W. Chen M. Cao Y. Lu W. Li X. (2024). The retinal pigment epithelium: functions and roles in ocular diseases. Fundam. Res. 4, 1710–1718. 10.1016/j.fmre.2023.08.011 39734536 PMC11670733

[B87] Warburton S. Davis W. E. Southwick K. Xin H. Woolley A. T. Burton G. F. (2007). Proteomic and phototoxic characterization of melanolipofuscin: correlation to disease and model for its origin. Mol. Vis. 13, 318–329. 17392682 PMC2642915

[B88] Wasmeier C. Hume A. N. Bolasco G. Seabra M. C. (2008). Melanosomes at a glance. J. Cell Sci. 121, 3995–3999. 10.1242/jcs.040667 19056669

[B89] Wavre-Shapton S. T. Tolmachova T. Lopes Da Silva M. Futter C. E. Seabra M. C. (2013). Conditional ablation of the choroideremia gene causes age-related changes in mouse retinal pigment epithelium. PLoS One 8, e57769. 10.1371/journal.pone.0057769 23460904 PMC3584022

[B90] Wavre-Shapton S. T. Meschede I. P. Seabra M. C. Futter C. E. (2014). Phagosome maturation during endosome interaction revealed by partial rhodopsin processing in retinal pigment epithelium. J. Cell Sci. 127, 3852–3861. 10.1242/jcs.154757 25074813 PMC4150067

[B91] Weil D. Blanchard S. Kaplan J. Guilford P. Gibson F. Walsh J. (1995). Defective myosin VIIA gene responsible for Usher syndrome type 1B. Nature 374, 60–61. 10.1038/374060a0 7870171

[B92] Williams D. S. (2008). Usher syndrome: animal models, retinal function of usher proteins, and prospects for gene therapy. Vis. Res. 48, 433–441. 10.1016/j.visres.2007.08.015 17936325 PMC2680226

[B93] Wolkow N. Li Y. Maminishkis A. Song Y. Alekseev O. Iacovelli J. (2014). Iron upregulates melanogenesis in cultured retinal pigment epithelial cells. Exp. Eye Res. 128, 92–101. 10.1016/j.exer.2014.09.010 25277027 PMC4252790

[B94] Wong W. L. Su X. Li X. Cheung C. M. Klein R. Cheng C. Y. (2014). Global prevalence of age-related macular degeneration and disease burden projection for 2020 and 2040: a systematic review and meta-analysis. Lancet Glob. Health 2, e106–e116. 10.1016/S2214-109X(13)70145-1 25104651

[B95] Zadlo A. Rozanowska M. B. Burke J. M. Sarna T. J. (2007). Photobleaching of retinal pigment epithelium melanosomes reduces their ability to inhibit iron-induced peroxidation of lipids. Pigment. Cell Res. 20, 52–60. 10.1111/j.1600-0749.2006.00350.x 17250548

